# S2. Therapeutic strategies based on overcoming immune resistance mechanisms within the melanoma tumour microenvironment

**DOI:** 10.1186/2051-1426-2-S2-I1

**Published:** 2014-03-12

**Authors:** T Gajewski

**Affiliations:** 1 University of Chicago, Department of Pathology, Chicago, IL, USA

## 

Two major categories of melanoma metastases have been observed based on gene expression profiling and confirmatory assays. One subgroup of patients has an inflamed phenotype that includes expression of chemokines, T cell markers, and other immunoregulatory factors. In contrast, the other major subset lacks this phenotype and appears to display immune “exclusion”. The optimal immunotherapeutic intervention to gain clinical benefit may be distinct in these two global subsets. The T cell-inflamed tumor microenvironment subset contains the highest expression of negative regulatory factors, including PD-L1, IDO, and FoxP3^+^ Tregs, and clear evidence for T cell-intrinsic anergy has also emerged. In addition, the mechanism of induction of these inhibitory mechanisms has been elucidated—PD-L1 and IDO are induced by IFN-γ, and Tregs are largely recruited by the chemokine CCL22, both being produced by activated CD8^+^ effector T cells. Preclinical experiments have confirmed a critical role for all 4 of these mechanisms in limiting anti-tumor T cell efficacy in vivo, giving candidate treatment strategies for translation back into the clinic. These include anti-PD-1/PD-L1 mAbs, IDO inhibitors, and approaches to deplete CD25^+^ Tregs and/or reverse anergy. The presence of multiple inhibitory mechanisms in the same tumor microenvironment argues that combination therapies may be advantageous to overcome compensatory effects. Preclinical data indicated synergy between anti-CTLA-4 +/- anti-PD-L1 +/- IDO inhibition. The mechanism of synergy is striking, as it correlates with a marked improvement of IL-2 production and proliferation of tumor-infiltrating CD8^+^ T cells. Clinical translation of these combination immunotherapies is promising and ongoing. In contrast to the T cell-inflamed melanomas, a new paradigm may be needed to promote de novo inflammation in cases of the non-T cell-infiltrated tumor microenvironment. Natural innate immune sensing of tumors appears to occur via the host STING pathway, type I IFN production, and cross-priming of T cells via CD8α^+^ DCs. New strategies are being developed to engage or mimic this pathway as a therapeutic endeavor.

